# Obtaining super-resolved images at the mesoscale through super-resolution radial fluctuations

**DOI:** 10.1117/1.JBO.29.12.126502

**Published:** 2024-12-24

**Authors:** Mollie Brown, Shannan Foylan, Liam M. Rooney, Gwyn W. Gould, Gail McConnell

**Affiliations:** a University of Strathclyde, Department of Physics, Glasgow, United Kingdom; b University of Strathclyde, Strathclyde Institute of Pharmacy and Biomedical Sciences, Glasgow, United Kingdom

**Keywords:** super-resolution, Mesolens, microscopy, diffraction-limited

## Abstract

**Significance:**

Current super-resolution imaging techniques allow for a greater understanding of cellular structures; however, they are often complex or only have the ability to image a few cells at once. This small field of view (FOV) may not represent the behavior across the entire sample, and manual selection of regions of interest (ROIs) may introduce bias. It is possible to stitch and tile many small ROIs; however, this can result in artifacts across an image.

**Aim:**

The aim is to achieve accurate super-resolved images across a large FOV (4.4×3.0  mm).

**Approach:**

We have applied super-resolution radial fluctuations processing in conjunction with the Mesolens, which has the unusual combination of a low-magnification and high numerical aperture, to obtain super-resolved images.

**Results:**

We demonstrate it is possible to achieve images with a resolution of 446.3±10.9  nm, providing a ∼1.6-fold improvement in spatial resolution, over an FOV of 4.4×3.0  mm, with minimal error, and consistent structural agreement.

**Conclusions:**

We provide a simple method for obtaining accurate super-resolution images over a large FOV, allowing for a simultaneous understanding of both subcellular structures and their large-scale interactions.

## 1 Introduction

The spatial resolution achievable in light microscopy discerns the smallest distance at which two individual structures can be resolved. This can be approximated using Rayleigh resolution limit,^1^ r, and is dependent on the numerical aperture (NA), of the lens used Eq. (1)

$$r=\frac{0.61\lambda }{\mathrm{NA}}.$$ (1)

Super-resolution microscopy refers to the group of techniques that surpass this limit to achieve higher resolutions than theoretically possible.^2^ Although these techniques allow a greater understanding of the cellular structure through the resolution of finer structures, there are often limitations associated. Methods such as stimulated emission depletion microscopy^3^ and structured illumination microscopy^4^^,^^5^ can significantly increase the resolution but require complex hardware. Single-molecule localization microscopy (SMLM) methods such as photoactivated localization microscopy^6^^,^^7^ and stochastic optical reconstruction microscopy^8^ are accessible with more conventional microscopes, but these methods require specialized fluorophores, with distinct on and off states. Large datasets capturing these individual states can be reconstructed to produce a super-resolved image, but the fluorophores can be expensive or inaccessible and thousands of images are necessary for reconstruction.^9^^,^^10^

In addition, for both shaped illumination super-resolution and SMLM techniques, a high NA lens is needed, and hence, the field of view (FOV) is often small, typically less than 100×100  μm^10^^,^^11^ sampling information from only a small number of cells in a single image. As such, manually selected regions of interest (ROIs) may introduce bias and may not be representative of the wider specimen. Stitching and tiling methods have been applied to produce super-resolved images over larger fields,^12^^,^^13^ but this computational approach can introduce artifacts where the edges of the tiles are poorly matched or where there is inconsistent illumination or fluorescence across separate tiles. Alternative methods using chip-based illumination have also been reported, achieving resolutions of up to 70 nm, but again, the FOV is restricted to 0.5×0.5  mm.^14^

In recent years, computational methods to produce super-resolved images have been developed that do not require complex equipment or labeling techniques. These methods use a similar principle to SMLM to reconstruct super-resolved images from the intensity fluctuations within diffraction-limited datasets.^15^^–^^18^ One of the most established methods is super-resolved radial fluctuation (SRRF).^15^ SRRF utilizes both the spatial and temporal information available within standard diffraction-limited widefield microscopy datasets to obtain super-resolved images by following similar principles as SMLM reconstruction without the need for specialized fluorophores. By measuring the radial symmetry (radiality) of the intensity surrounding each pixel in an image, SRRF works to calculate the probability of each pixel containing fluorescence. If a pixel has a uniform radial symmetry (i.e., high radiality), then it is likely fluorescent, whereas pixels with low radiality may be attributed to noise or spurious non-fluorescent events. The correlation of each point throughout the stack is processed using a selected temporal analysis algorithm to create a final SRRF image that considers both the spatial and temporal information. There are a variety of adjustable parameters that allow for the refinement of any SRRF analysis. Of these, two are particularly impactful on the work shown here and should be noted—the radiality magnification and ring radius. The ring radius determines the radius of the ring which the radiality measurements are taken from and should be adjusted depending on the density of the datasets. When SRRF calculates the radiality for each pixel, it can do this on a singular pixel, or more typically, it magnifies each pixel into a grid of n×n subpixels, where n is the magnification, and performs this radiality measurement on each sub-pixel. This often leads to resolution up to fivefold higher in the final reconstruction;^16^ however, increasing the pixels within the image also in turn increases the size of the image file.

Following SRRF processing it is important to verify the accuracy of the super-resolution reconstructions. This is achieved using two primary methods. The first is analysis with super-resolution quantitative image rating and reporting of error locations (SQUIRREL),^19^ to both map the accuracy across the image and quantify the error within the super-resolution images using resolution scaled Pearson’s coefficient (RSP) and resolution scaled error (RSE). The second method is through measuring the resolution of the final super-resolution image with image decorrelation analysis.^20^

SRRF has been widely used for a variety of biological applications, with studies reporting an increase in spatial resolution from 235 to <100  nm in the cell wall of xylem from Douglas fir trees, although over a limited field with only a few cells analyzed per image.^21^ This approximate twofold improvement in resolution is consistent across other work using SRRF, including the quantification and localization of azurophilic granules in neutrophil leukocytes,^22^ where the resolution of raw data was increased from 160 nm to better than 100 nm using SRRF, but again, only a few cells were captured in each image.^22^

**Fig. 1:**
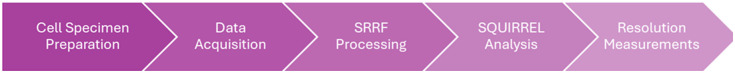
Overview of the processing stages in obtaining super-resolved Mesolens images.

Here, we demonstrate that by applying SRRF, in conjunction with diffraction-limited widefield Mesolens imaging, as seen in Fig. 1, it is possible to obtain super-resolved images over a large multi-millimeter FOV through the analysis of intensity fluctuations across widefield Mesolens images.

The unusual combination of low magnification and high NA from the Mesolens (4×, 0.47 NA) enables imaging over a FOV of 4.4×3.0  mm, with a lateral resolution of up to 700 nm^23^ and the ability to capture over 700 cells per image.^24^ Using SRRF, we aimed to improve the lateral resolution as far as possible while retaining the large FOV for super-resolved imaging of larger cell populations than are traditionally imaged using SRRF microscopy.

## 2 Materials and Methods

### 2.1 Cell Specimen Preparation

HeLa cells (CVCL_0030v, ATCC) were cultured at 37°C, 5% ${\mathrm{CO}}_{2}$, in Dulbecco’s modified Eagle medium (DMEM) (11960044, Thermo Fisher, Waltham, Massachusetts, United States) containing 1% penicillin–streptomycin (P4458-100ML, Merck, Rahway, New Jersey, United States), 1% l-glutamine (392-0441, VWR), and 10% fetal bovine serum (10500064, Gibco, Waltham, Massachusetts, United States). Coverslips were coated with a 1:500 dilution of fibronectin bovine plasma (F1141-1MG, Merck) and incubated for 24 h prior to fixation.

Coverslips were fixed with 4% paraformaldehyde (PFA) (16005, Merck) at 37°C for 15 min, before being blocked and permeabilized for 30 min at 37°C in an immunofluorescence (IF) buffer comprising 2.5% goat serum (ab7481, Abcam, Cambridge, United Kingdom) and 0.3% TritonX-100 (X100, Merck) in phosphate-buffered saline (PBS), labeled using anti-tubulin rat monoclonal conjugated with Alexa Fluor 488 (ab195883, Abcam) at a dilution of 1:100 in IF buffer and incubated at 4°C for 24 h in a dark culture dish with stable humidity. The samples were then mounted using ProLong Glass (P36980, Thermo Fisher) to reduce photobleaching during prolonged imaging and left to set for at least 24 h prior to imaging.

For non-filamentous structures, a stable cell line of HA-GLUT4-GFP expressing 3T3-L1 cells was previously generated in the lab^25^ plated on 18-mm-diameter coverslips and grown in DMEM (52100047, Gibco) supplemented with 10% new-born calf serum (26010066, Gibco), 5% l-glutamine, 5% penicillin–streptomycin and maintained at 37°C, and 10% ${\mathrm{CO}}_{2}$. Following differentiation into adipocyte^26^ cells were put on ice, washed once with ice-cold 1X PBS, and fixed in 4% PFA for 30 min at room temperature. Coverslips were washed three times with 1X PBS before mounting on a slide with ProLong Glass. Specimens were allowed to set fully prior to imaging.

### 2.2 Mesolens Imaging and Data Acquisition

**Fig. 2:**
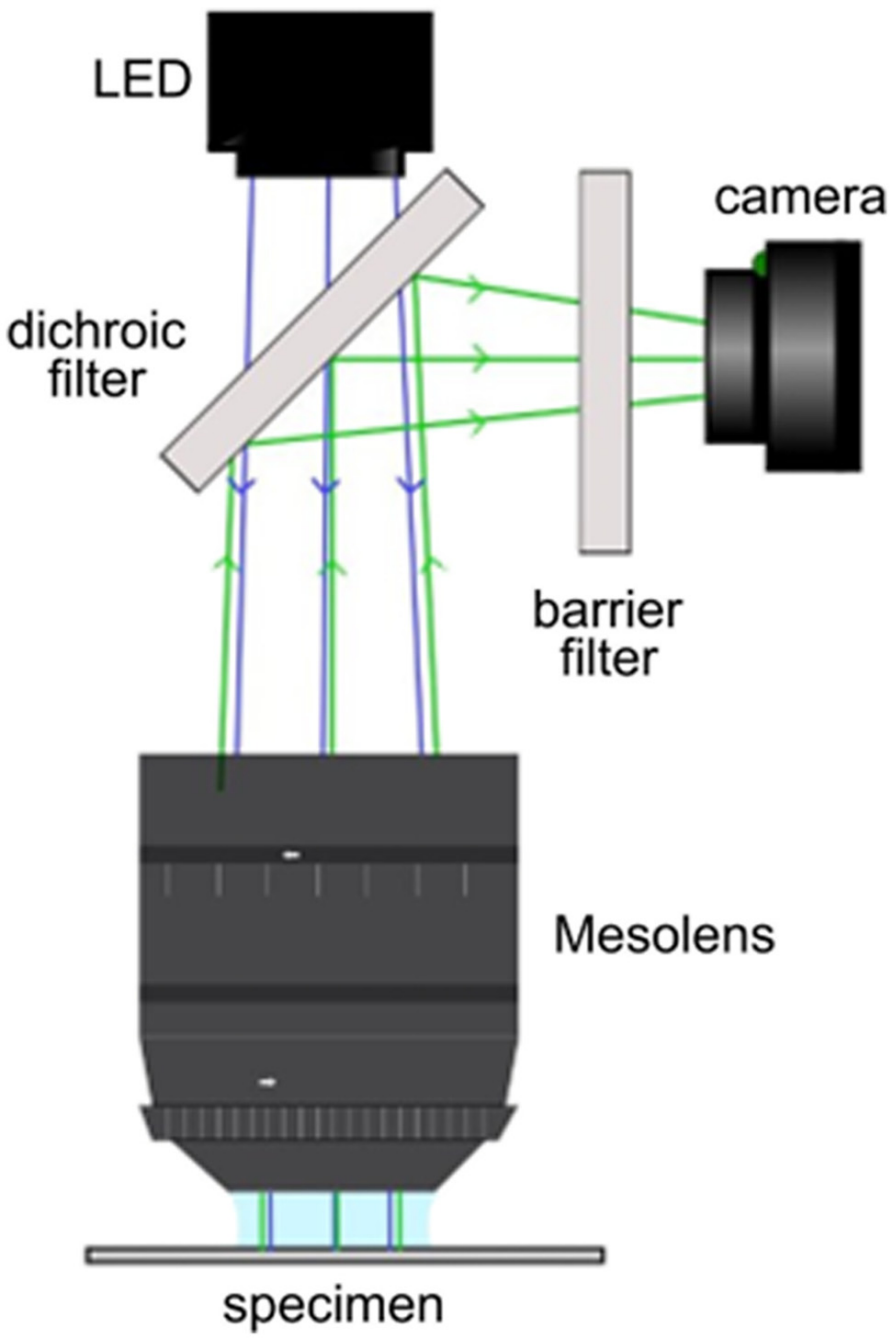
Schematic diagram of the widefield epifluorescence Mesolens system.

Diffraction-limited images were obtained with the Mesolens using the widefield fluorescence illumination modality, a diagram of this system setup is shown in Fig. 2. Specimens were illuminated using a 490±20  nm light-emitting diode (LED) sourced from a multi-wavelength illuminator (pE-4000, CoolLED, Andover, United Kingdom), and fluorescence emission was detected at 525±25  nm. The maximum power of the excitation source at the specimen plane was 35.7±0.1  mW. Imaging was carried out with the Mesolens correction collars set for water immersion to minimize spherical aberration. To ensure statistically independent images for SRRF processing, a time-lapse series of the fixed samples was taken to obtain 40 images, with the interval between image capture typically set to 2 to 3 s as the samples are fixed. Other works utilizing SRRF can use up to hundreds of individual images^27^ to produce the final reconstruction; however, due to the size of the Mesolens image datasets and the computational restrictions this entails the datasets here are limited to 40 images for SRRF processing. This restricted dataset may reduce the ability of SRRF processing to suppress noise to avoid this exposure time was typically kept above 1500 ms when imaging, as longer exposure times would result in a higher SNR.

To ensure the diffraction-limited widefield Mesolens images captured are of the highest resolution, images are captured using a camera with a chip-shifting sensor (VNP-29MC, Vieworks, Anyang-si, South Korea). This shifting moves the 29-megapixel chip in a 3×3 array, giving an image resolution of 19,728×13,152  pixels for a total 259.5-megapixel capture. Over the 4.4×3.0  mm FOV, this results in a 226-nm pixel size—satisfying Nyquist sampling.^28^ The acquisition of one full FOV image using the sensor shifting camera took 13.5 s when using a 1500-ms exposure. When including the additional time for the transfer of image data to the computer, and the interval between captures, imaging of a 40-image stack typically took ∼22  min.

### 2.3 SRRF Processing

The diffraction-limited widefield Mesolens images were opened within Fiji.^29^ To avoid the possibility of introducing any artifacts no preliminary processing was carried out, instead using the Nano-J SRRF plugin^16^ the raw images can be processed. There were restrictions on the parameters that can be used when processing Mesolens images due to the unconventional file size of the datasets, and as such the parameters used vary slightly from the default values. The ring radius was set to 1.90—when carrying out quantification of the image resolution this was found to result in the highest resolution while also allowing for ease of processing. The radiality magnification was reduced so that the large datasets could be processed. This was kept at two as it allowed for SRRF processing of intact Mesolens datasets. If the magnification was increased further processing could only be carried out on small ROIs, which would introduce stitching and tiling artifacts. For the temporal analysis, temporal radiality pairwise product mean (TRPPM) was selected as the fluorophores used do not blink but instead have slight intensity fluctuations which TRPPM is best suited to process. Using TRPPM over alternative options such as temporal radiality average is also beneficial as it provides additional noise suppression, which is beneficial given the limitations on the size of the datasets used.^15^ Any unmentioned parameters remained at the default value. The typical processing time of one dataset was ∼95  min, including opening the dataset within Fiji. The computational power available was limited, and this figure could be greatly reduced in further iterations.

### 2.4 SQUIRREL Analysis and Resolution Measurements

Following SRRF processing, the accuracy of the output image was first assessed using SQUIRREL. Due to the size of the processed SRRF images, SQUIRREL analysis could not be conducted on the intact image. As there is no radiality component within SQUIRREL, the image was split into tiles and later recombined following processing without the introduction of artifacts on the edge of each tile. Macros were used within Fiji to split both the reference image and super-resolution SRRF image into tiles of equal sizes, with the required diffraction-limited reference image^19^ taken as the first diffraction-limited widefield Mesolens image from each stack. From here, it was possible to perform SQUIRREL analysis on each of the matching tile pairs, with the RSF estimated through optimization each time, and the individual SQUIRREL error tiles could then be stitched^30^ into one complete error map. The individual RSP and RSE values calculated for each tile were averaged to determine the value for the complete image, with the error taken as the standard deviation. RSP measures the Pearson correlation coefficient among the images on a normalized scale between −1 and 1, with one being identical images. This acts as a measurement of the structural correlation among images and is independent of intensity. RSE is an intensity-dependent measurement of the root-mean-square error among images, where lower values indicate better agreement between the raw image and the super-resolution equivalent. For ease of comparison among datasets, the RSE value was normalized against the total range of possible values. Typically, each Mesolens image is split into 36 tiles for processing, and SQUIRREL analysis of each pair of tiles takes ∼1  h.

The resolution was measured using image decorrelation analysis^20^ Fiji plugin. Due to restrictions imposed by the large Mesolens file size, the measurements were carried out on n=3 ROIs from across the field of each super-resolution reconstruction, and the final resolution for each image is an average of these ROIs. Each ROI was cropped from the original super-resolution SRRF Mesolens images, with no additional image processing prior to this stage to ensure an accurate measurement. The ROIs were opened within Fiji and then processed using image decorrelation analysis and the default settings. The resultant plots show the decorrelation curves of the ROI, along with the calculated cutoff frequency, $k_{c}$, and the amplitude of the local maximum of the decorrelation function, $A_{0}$. The achievable resolution presented here is an average of N=9 ROIs, across N=3 tubulin-labeled datasets—again the error is taken as the standard deviation.

## 3 Results

### 3.1 Applications to Filamentous Structures

**Fig. 3:**
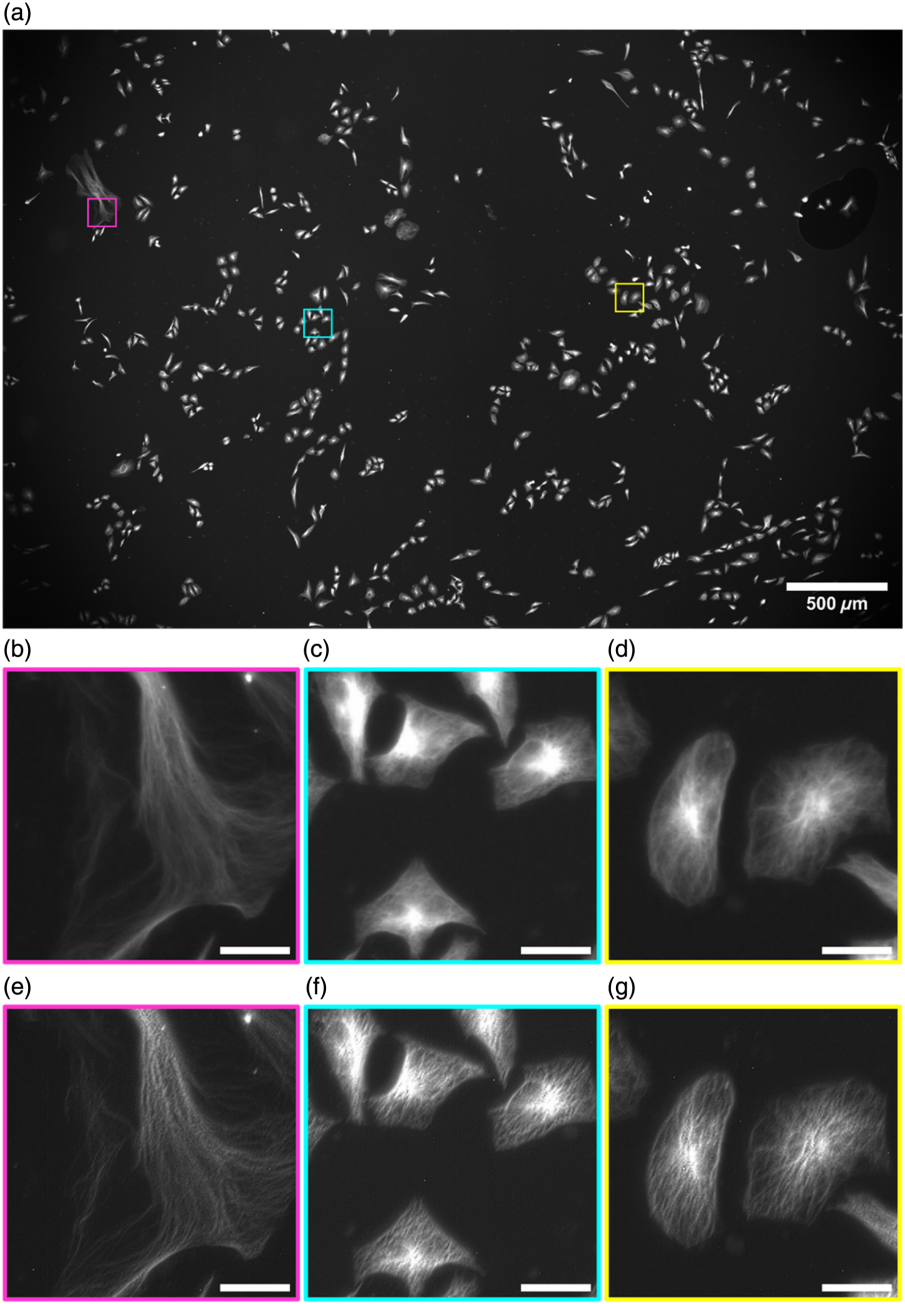
Complete Mesolens FOV showing the SRRF-processed image of the tubulin in HeLa cells labeled with AF488 with three highlighted ROI: magenta, cyan, and yellow (a). A digital zoom of the raw widefield epifluorescence diffraction limited Mesolens image is shown for each ROI (b)–(d), with a digital zoom of the improved ROI following SRRF processing (e)–(g). Scale bars are 30  μm for all ROIs.

Figure 3(a) shows a full Mesolens FOV image of tubulin-labeled HeLa cells, following SRRF processing, demonstrating the scale and the volume of cells captured per image, along with the absence of any stitching and tiling artifacts across the entire FOV of super-resolved reconstruction. Digital zooms of ROI are highlighted to demonstrate the improvement in contrast and resolution between the original Mesolens images [Figs. 3(b)–3(d)] and the corresponding SRRF equivalent [Figs. 3(e)–3(g)]. Between each of the corresponding ROI pairs, there is a clear improvement in image clarity and spatial resolution, allowing for a level of detail not previously visualized across the mesoscale. No additional image processing was carried out on either set of images.

**Fig. 4:**
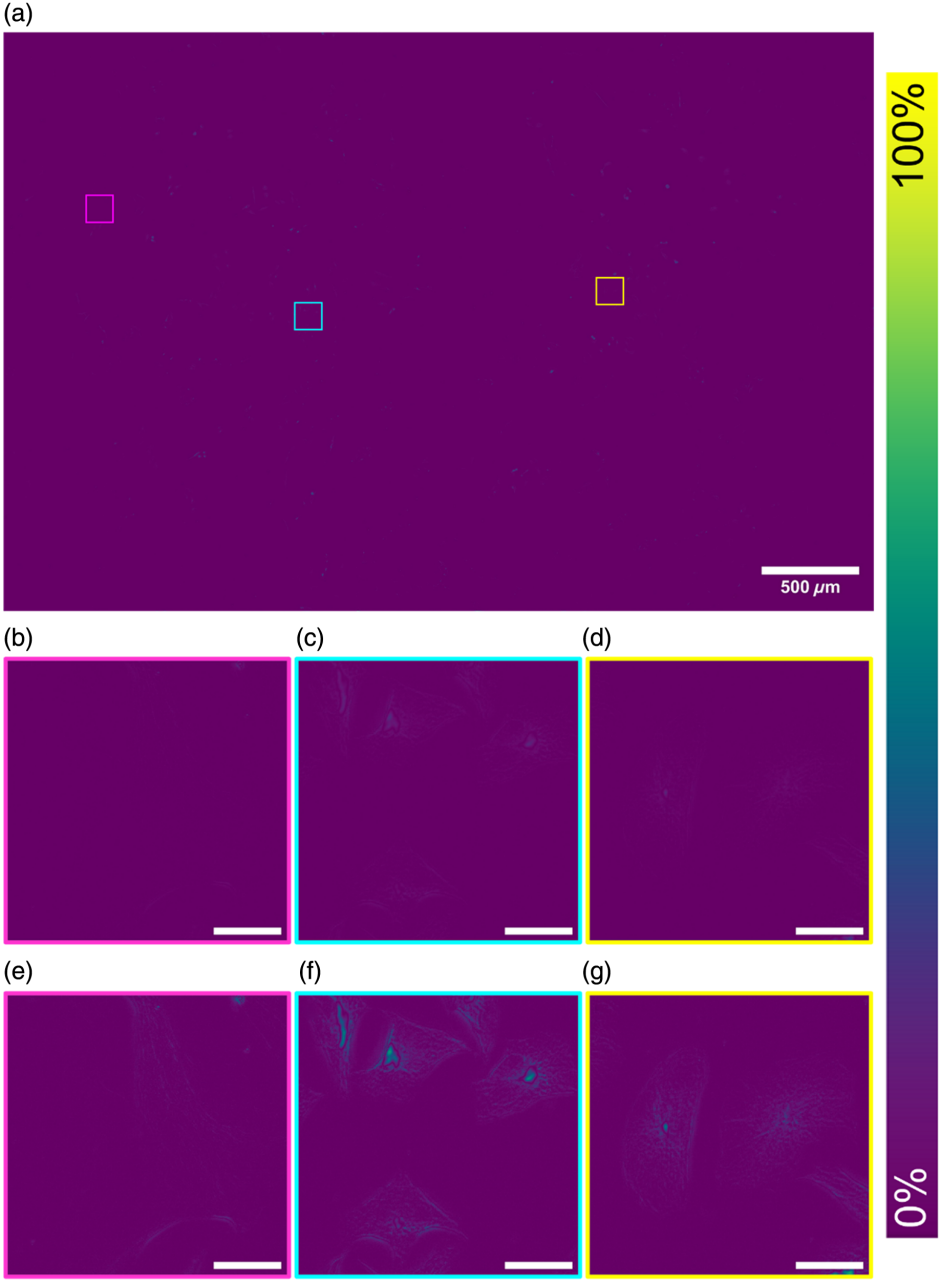
Complete reconstructed SQUIRREL error map showing the areas of agreement between the raw widefield epifluorescence diffraction limited Mesolens image and the SRRF reconstruction (a). In the middle row, a digital zoom of the error map is displayed for each ROI (b)–(d), and in the bottom row, a contrast-adjusted ROI highlighting the areas of error (e)–(g). Scale bars are 30  μm for all ROIs.

SQUIRREL analysis on the dataset shown in Fig. 3 is presented in Fig. 4. These data show there is minimal error and high levels of agreement between the original and the super-resolution images across the full FOV [Fig. 4(a)]. The same ROIs in Fig. 3 are shown with SQUIRREL processing in Figs. 4(b)–4(d). These show the areas of disagreement between the raw Mesolens image and the super-resolution equivalent. These ROIs highlight that this low error and high agreement is continuous across both the full FOV and the individual regions and that although there are areas of disagreement across the map, the disagreement is small. Figures 4(e)–4(g) show digital zooms of a contrast-adjusted error map. The image was adjusted using contrast-limited adaptive histogram equalization (CLAHE) with the default parameters (block size = 127, histogram bins = 256, maximum slope= 3.0, mask = none, and fast = false)^31^ to highlight the areas of discrepancy. We note these discrepancies are largely confined to areas where the image is saturated.

For the dataset shown in Figs. 3 and 4, this high fidelity among images is confirmed by the RSP value of 0.989±0.004. RSP is a normalized value between −1 and 1, with one representing structurally identical images. The value shown here indicates high structural agreement, and minimal artifacts between both images, alongside the intensity-dependent RSE result of 1.13%±0.23%. The RSE value presented has been normalized against the total intensity range in the SQUIRREL error map, allowing for simplified comparisons among different datasets. The resolution of this super-resolution reconstruction in Fig. 3(a) was also measured from three ROI at 442.9 nm [see Figs. S1(a)–1(c) in the Supplementary Material]. A significant improvement in image resolution surpasses the theoretical maximum resolution achievable on the Mesolens—providing super-resolved images.

To calculate an average resolution measurement achieved using this method, measurements were repeated for three biological repeats of HeLa cells where tubulin was labeled with an AF488 antibody conjugate. There was an average RSP value of 0.991±0.006 and an average RSE value of 0.81%±0.30%, with consistently low error rates and high agreement across different images. An average resolution was also recorded using three ROI from each sample and measured at 446.3±10.9  nm (see Fig. S1 in the Supplementary Material).

**Fig. 5:**
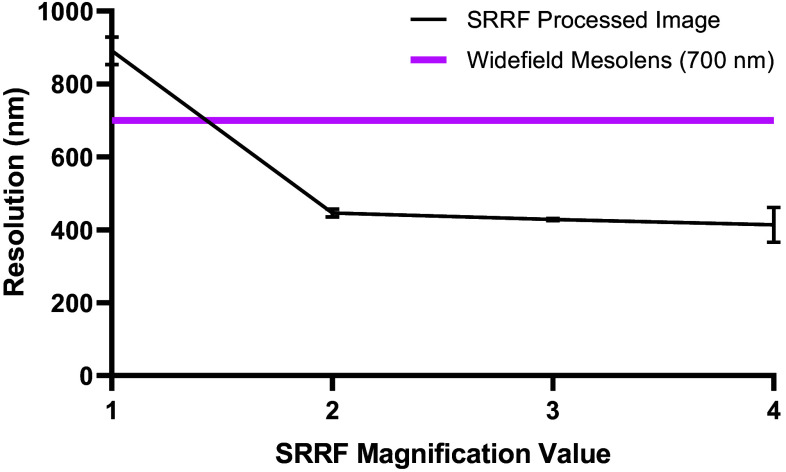
Image resolution measured using image decorrelation analysis, for increasing SRRF magnification, comparative to the previously shown Mesolens resolution of 700 nm.^23^ Error bars represent the standard deviation.

Due to the size of the Mesolens data, it is currently not feasible to increase the SRRF magnification above 2 on raw data without surpassing the maximum allowed file size for processing. For higher magnification values, SRRF processing is possible on ROIs only, so a magnification of 2 is used here to allow for the processing of raw Mesolens data without the addition of stitching and tiling artifacts. To demonstrate that using a higher magnification would have minimal impact on image resolution, SRRF was carried out on the same ROIs as shown in Fig. 3, using increasing magnification and the average resolution across these ROIs for each magnification was calculated, as shown in Fig. 5.

### 3.2 Applications to Non-Filamentous Structures

**Fig. 6:**
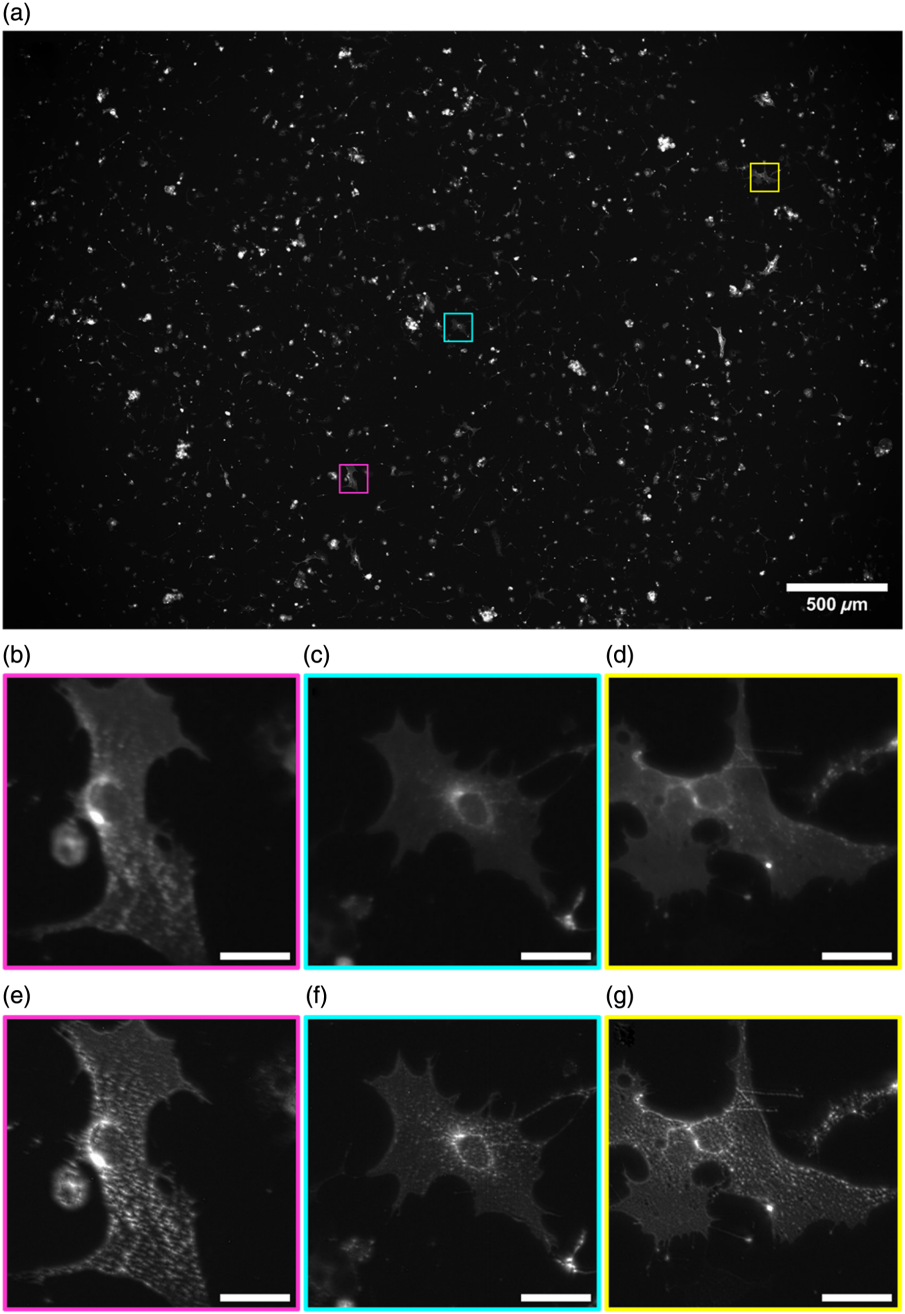
Complete Mesolens FOV showing the SRRF processed image of the GLUT-4 in 3T3-L1 adipocytes expressing GFP, with three highlighted ROI: magenta, cyan, and yellow (a). A digital zoom of the raw widefield epifluorescence diffraction limited Mesolens image is shown for each ROI (b)–(d), with a digital zoom of the improved ROI following SRRF processing below (e)–(g). Scale bars are 30  μm for all ROIs.

To demonstrate this method is not confined to filamentous structures, Fig. 6 shows a full Mesolens FOV capture of green fluorescent protein (GFP) tagged GLUT4 glucose transporters in 3T3-L1 fibroblasts following SRRF processing, with digitally magnified ROIs to again show the improvement in the SRRF reconstruction from the original widefield epifluorescence diffraction limited Mesolens image. Figure 6(a) shows the entire FOV of super-resolved reconstruction, and the intact cells captured again without any stitching and tiling artifacts. Digital zooms of ROIs demonstrate the improvement in contrast and resolution between the original Mesolens images [Figs. 6(b)–6(d)] and SRRF equivalent [Figs. 6(e)–6(g)]. Again, between each of the ROI pairs, there is a clear improvement in resolution and image clarity, allowing for clearer visualization of the GLUT4 molecules throughout the cell interior. GLUT4 is sequestered within multiple different intracellular compartments in the absence of insulin,^25^^,^^32^ and the images shown here provide a clear illustration of this distribution. The resolution of this image was measured from three ROIs at 431.6±9.8  nm (see Fig. S2 in the Supplementary Material), which again surpasses the maximum optical resolution theoretically possible on the Mesolens, obtaining super-resolved images.

**Fig. 7:**
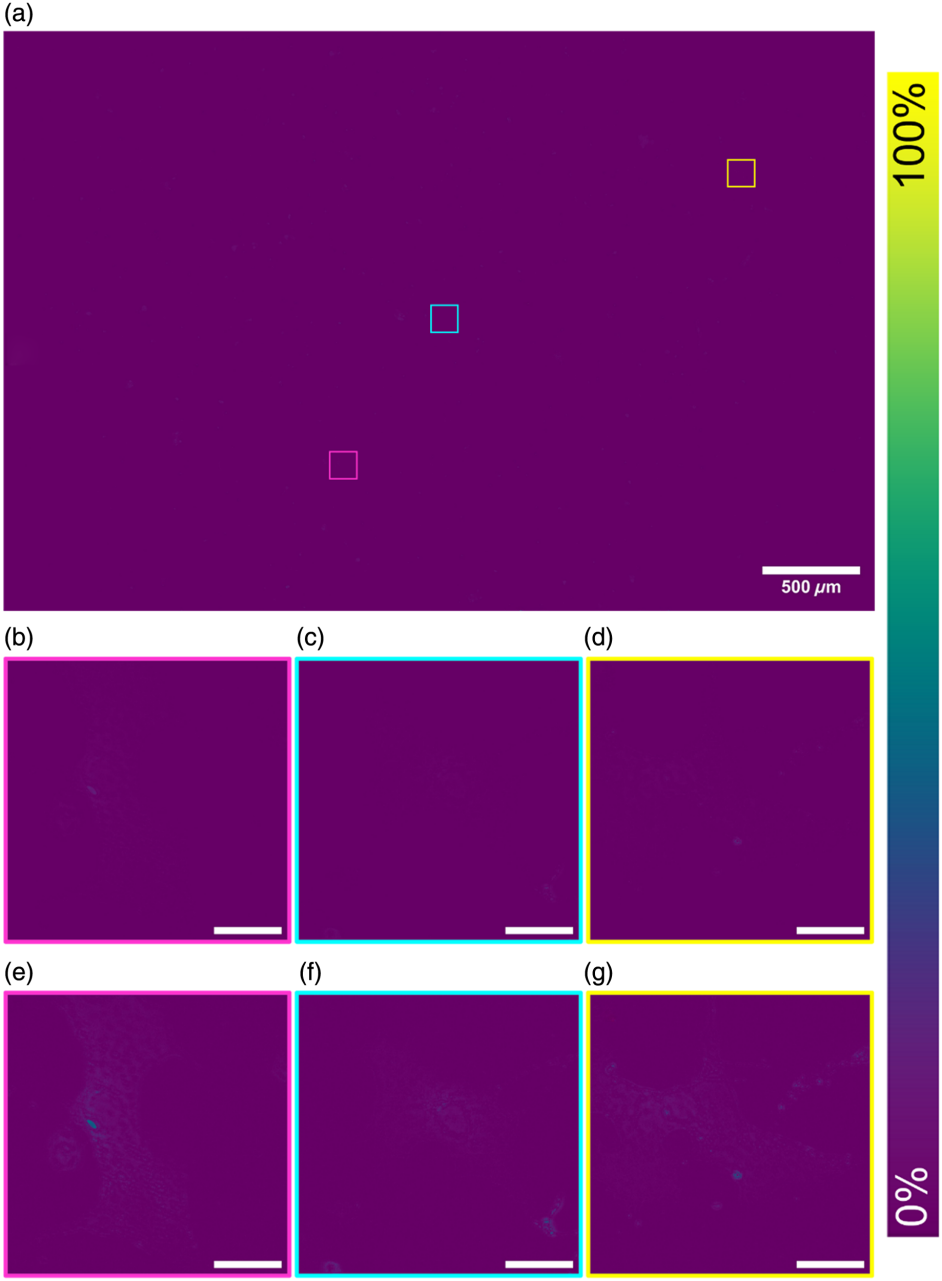
Reconstructed SQUIRREL error map showing the areas of agreement between the raw widefield epifluorescence diffraction limited Mesolens image and the SRRF reconstruction with highlighted ROI: magenta, cyan, and yellow (a). A digital zoom of the error map is displayed for each ROI (b)–(d), with a contrast-adjusted ROI highlighting the areas of error within each ROI below (e)–(g). Scale bars are 30  μm for each ROI.

SQUIRREL analysis on this dataset is shown in Fig. 7. There is again minimal error and high levels of agreement between the raw widefield epifluorescence diffraction limited Mesolens image and the SRRF reconstruction throughout across the full FOV [Fig. 7(a)]. Digital zooms of the ROIs [Figs. 7(b)–7(d)] highlight that there is some error present across the FOV, but it is minimal. Figures 7(e)–7(g) show digital zooms of a contrast-adjusted error map. The image was adjusted using CLAHE with the default parameters (block size = 127, histogram bins = 256, maximum slope = 3.0, mask = none, and fast = false)^31^ to better highlight the areas of discrepancy. For this dataset, the average RSP value was calculated as 0.993±0.003 and the RSE value as 0.68%±0.14%, again demonstrating the high fidelity and low levels of error between the original raw diffraction limited widefield Mesolens image and the SRRF processed reconstruction.

## 4 Discussion and Conclusion

Following SRRF processing, there is a clear visual improvement in spatial resolution and contrast, allowing for clearer visualization of the filamentous and non-filamentous structures imaged. Using the 446.3-nm resolution obtained from the SRRF (M=2) value for filamentous structures and a wavelength of 530 nm with Eq. (1), we can consider that the effective NA of the Mesolens has increased from 0.47 to ∼0.72, with no reduction in FOV.

The average resolution stated, 446.3±10.9  nm, is found from n=9 ROIs from across the FOV of three biological replicates. Due to these replicates and the low error found within this average, the resolution is taken as consistent across the entire FOV. Any aberrations within the widefield Mesolens imaging field have been shown previously to be minimal,^23^ and the uniformity holds across the entire FOV.

The error maps show that there is minimal error and that the SRRF images are in high agreement with the original Mesolens images. It is possible to see that these are largely within the high-intensity areas where it is difficult to visibly discern fine structures within the original images. This low error is also validated by the average RSP and RSE values, which indicates there is high fidelity between both images. This is further illustrated by the resolution measurement, which shows a notable improvement following SRRF, surpassing the resolution limit for the Mesolens and achieving super-resolved images.

As seen in Fig. 6, this method is not limited to filamentous structures and can be used to accurately improve the resolution of other, non-filamentous cellular structures, demonstrating its capabilities. The resolution of the 3T3-L1 fibroblasts expressing GFP labeled GLUT4 was measured at 431.6±9.8  nm, which is higher than the achievable resolution stated previously. This resolution of 446.3±10.9  nm is calculated from three ROIs from three biological replicates (compared with the three ROIs from one biological replicate here) and as such provides a theoretically achievable resolution, which may vary depending on the samples and the imaging conditions used. The intracellular localization of GFP-tagged GLUT4 to vesicles within the cell is clearly revealed using this approach.

Using SRRF in conjunction with the Mesolens allows for an increased understanding of many cellular structures, with an improvement in resolution comparable to other work utilizing SRRF. However, using this method circumvents any potential artifacts introduced by stitching and tiling or from manually selecting ROIs—providing a more accurate understanding of the entire field. It has a wide range of potential applications—the increase in spatial resolution would allow for more accurate co-localization studies to better determine the location and interaction of molecules throughout a cell population. The use of widefield epifluorescence imaging in obtaining these images is also less harsh to samples than other conventional super-resolution microscopy methods, allowing for preservation for repeated imaging.

Other work utilizing SRRF often uses higher magnification values to obtain super-resolved images.^21^^,^^27^^,^^33^ Increasing the magnification also increases the size of the image being processed, as each original pixel is split into a grid of smaller pixels, increasing the data contained within one image. This increase in image size limits the processing of intact Mesolens data to using a magnification of 2 as beyond this processing is only possible on ROIs. As seen in Fig. 5, there is a slight increase in the resolution achievable when increasing magnification beyond 2. However, carrying out processing on individual ROIs would introduce stitching and tiling artifacts, and the impact of this would outweigh any marginal improvements in resolution. If future work is able to circumvent the computational limitations currently in place, it may allow for the use of higher magnifications to provide an additional increase in resolution. The computational power available here also limits the processing time, particularly for error analysis using SQUIRREL, if this can be increased then processing time would be significantly reduced.

A recent SRRF successor, enhanced super-resolution radial fluctuations (eSRRF),^17^ expands upon the same analysis method to improve the accuracy of the super-resolution images, and further increase the achievable resolutions while also providing the capability to carry out 3D image reconstructions. However, due to the size of the Mesolens datasets used, it was deemed impractical to use eSRRF. It would be possible to split the Mesolens images into individual tiles and apply eSRRF to each tile; however, due to the radiality component of the method, the pixels at the edge of each tile could not be properly analyzed, with only a fraction of the radial information available. This would introduce artifacts and inconsistent intensities across the tile edges. Due to this, SRRF is used rather than eSRRF for this study. As with previous issues, if the computational limitations can be resolved then future work may be able to utilize eSRRF, achieve higher resolution results, and also unlock the potential to carry out 3D super-resolution mesoscale imaging.

Although both SRRF and its successor eSRRF are capable of live cell super-resolution reconstructions, due to the long acquisition time of one Mesolens image, 13.5 s when using a 1500 ms exposure time, and the current absence of an environmental imaging chamber this method is unsuitable for the imaging of fast cell processes. However, there may be scope for future super-resolution live-cell work on the Mesolens with new sensors with higher pixel numbers and faster chip-shifting.

In this work, we have demonstrated using SRRF in conjunction with diffraction-limited widefield Mesolens images, that it is possible to obtain super-resolved images at the mesoscale—achieving a resolution of 446.3 nm across a 4.4×3.0  mm FOV. With SQUIRREL analysis and error maps demonstrating consistent agreement between the original and SRRF processed images validating the accuracy of the reconstructions. This provides a comparatively cost-efficient and simple method for obtaining accurate super-resolution images over a large FOV, allowing for a simultaneous understanding of the subcellular structures alongside their large-scale interactions.

## Supplementary Material



## Data Availability

The data presented in this article are publicly available on request from the University of Strathclyde KnowledgeBase at https://doi.org/10.15129/1897cdca-4dae-4ab4-8a21-aec16c8820d5. This dataset includes raw images in .tiff format for all imaging with accompanying ROI sets.
