# Supplementary material for: Obtaining super-resolved images at the mesoscale through super-resolution radial fluctuations
Source: J Biomed Opt. 2024 Dec 24;29(12):126502. doi: 10.1117/1.JBO.29.12.126502 (PMC11667203; doi:10.1117/1.JBO.29.12.126502)
Supplement: Supplementary file 1 [file JBO_029_126502_SD001.pdf]

Obtaining super-resolved images at the mesoscale through Super-Resolution Radial Fluctuations

Mollie Brown, [a,*] Shannan Foylan,[b] Liam M. Rooney,[b] Gwyn W. Gould,[b] Gail McConnell[b]

[a]University of Strathclyde, Department of Physics, Glasgow, UK
[b]University of Strathclyde, Strathclyde Institute of Pharmacy and Biomedical Sciences, Glasgow, UK

Supplemental Information

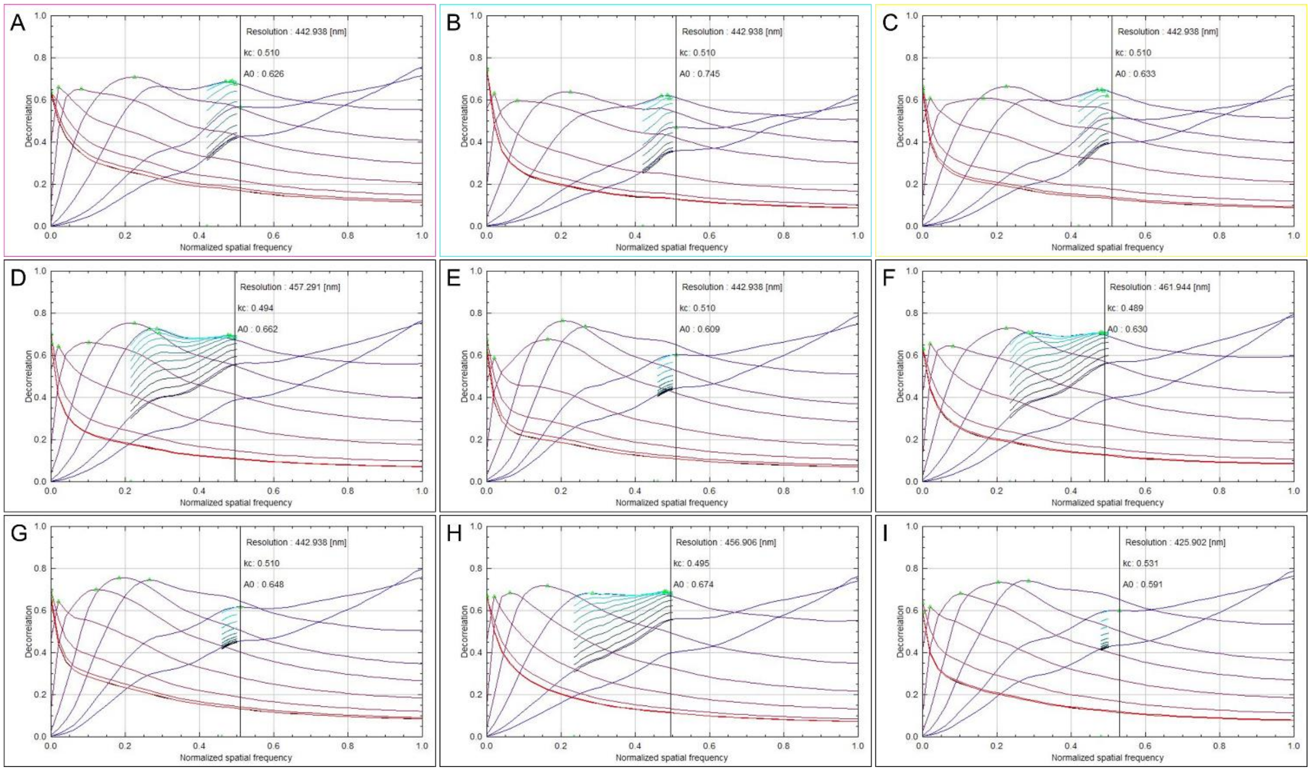

Figure S1: The plots of all decorrelation curves used to calculate the achievable resolution. Including the magenta, cyan and yellow ROI as shown in Figures 3 and 4 from biological replicate 1 (A-C), and the decorrelation curves calculated from three ROI from biological replicate 2 (D-F), and biological replicate 3 (G-I).

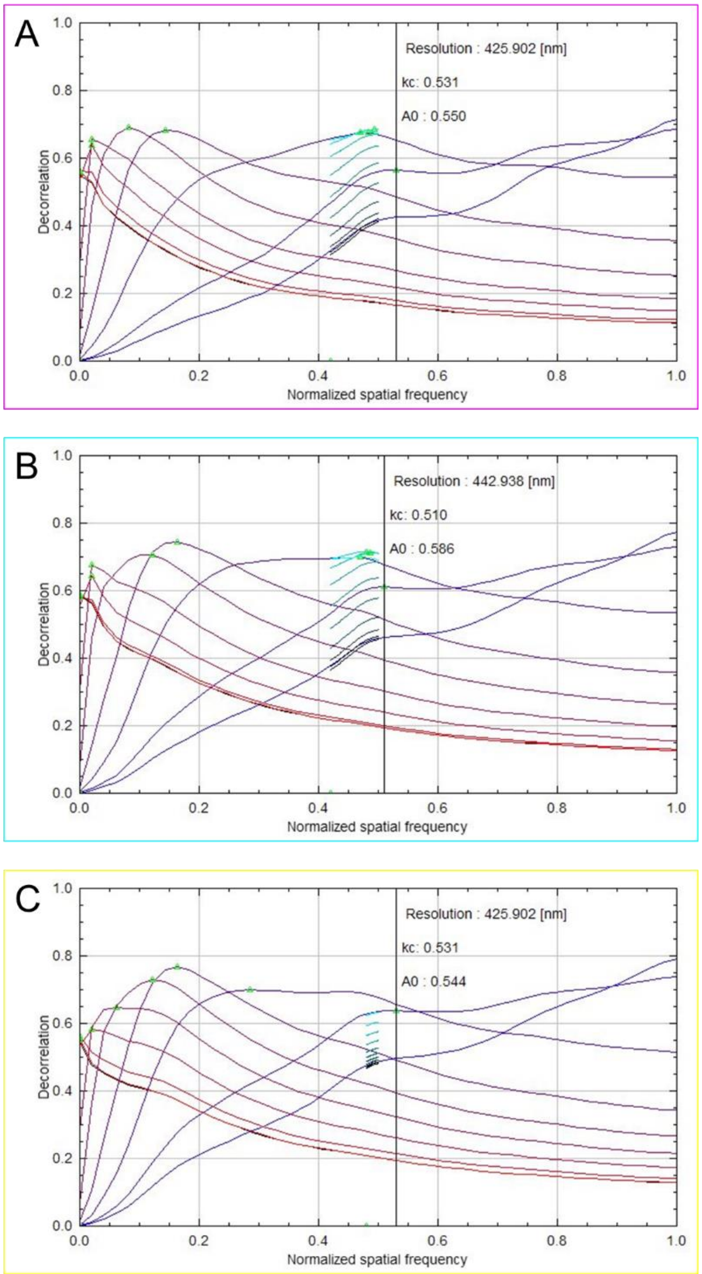

Figure S2: The plots of all decorrelation curves used to calculate the resolution of GFP-tagged GLUT4 glucose transporters in 3T3-L1 fibroblasts. A, B and C show the decorrelation curves from the magenta, cyan, and yellow ROIs as shown in figures 6 and 7.
